# De-MetaST-BLAST: A Tool for the Validation of Degenerate Primer Sets and Data Mining of Publicly Available Metagenomes

**DOI:** 10.1371/journal.pone.0050362

**Published:** 2012-11-26

**Authors:** Christopher A. Gulvik, T. Chad Effler, Steven W. Wilhelm, Alison Buchan

**Affiliations:** 1 Department of Microbiology, University of Tennessee, Knoxville, Tennessee, United States of America; 2 Department of Electrical Engineering and Computer Science, University of Tennessee, Knoxville, Tennessee, United States of America; University of Delaware, United States of America

## Abstract

Development and use of primer sets to amplify nucleic acid sequences of interest is fundamental to studies spanning many life science disciplines. As such, the validation of primer sets is essential. Several computer programs have been created to aid in the initial selection of primer sequences that may or may not require multiple nucleotide combinations (*i.e.,* degeneracies). Conversely, validation of primer specificity has remained largely unchanged for several decades, and there are currently few available programs that allows for an evaluation of primers containing degenerate nucleotide bases. To alleviate this gap, we developed the program De-MetaST that performs an *in silico* amplification using user defined nucleotide sequence dataset(s) and primer sequences that may contain degenerate bases. The program returns an output file that contains the *in silico* amplicons. When De-MetaST is paired with NCBI’s BLAST (De-MetaST-BLAST), the program also returns the top 10 nr NCBI database hits for each recovered *in silico* amplicon. While the original motivation for development of this search tool was degenerate primer validation using the wealth of nucleotide sequences available in environmental metagenome and metatranscriptome databases, this search tool has potential utility in many data mining applications.

## Introduction

PCR is one of the most fundamental and powerful molecular biology tools available. PCR primer sets that contain degenerate bases allow for the amplification of homologous sequences and have been used in various applications, including genetic diversity analyses (*e.g.,* [1]–[12]). Several software packages that use a nucleotide or amino acid alignment of the genetic target are available to aid in the initial development of degenerate primer sets (*e.g.*, Amplicon [13], CODEHOP [14]–[16], DEFOG [17], DePiCt [18], HYDEN [19], MAD-DPD [20], PhiSiGns [21], and Primaclade [22]). In addition, manual identification of conserved regions from aligned sequences generated using software such as ARB [23], ClustalX [24], and MEGA [25] is also common practice (*e.g.,* [26]–[31]). Once candidate primers are developed, thermodynamic properties and self-complementarity tests can be obtained using online tools (*e.g.*, OligoCalc [32]).

Despite the utility and common use of degenerate primers, there are no software programs specifically designed to facilitate validation of their specificity. The most common practice for initial validation of degenerate primers is by direct sequence analysis of PCR amplicons (*e.g.,* [33]–[37]). This can be both laborious and costly, and does not take advantage of the ever-increasing publicly available nucleotide data, including that derived from natural samples. In fact, environmental metagenomes and metatranscriptomes are especially attractive reference databases (*e.g.,* CAMERA [38] [http://camera.calit2.net/] and MG-RAST [http://metagenomics.anl.gov/]) to perform *in silico* tests *en masse* to identify sequences a degenerate primer set might amplify.

To address this gap in available bioinformatic tools, we have developed a program termed De-MetaST. This program accepts primers that are degenerate using a meta-genome and –transcriptome search tool to retrieve *in silico* PCR amplicons. When paired with BLAST [39], the output provides the most homologous sequences in GenBank for each recovered *in silico* amplicon. In this report, we provide an overview of the program and outline its utility as a tool to validate the specificity of degenerate primer sets. This program is designed to be user-friendly for non-bioinformatics specialists and is publicly available; as are screencast video tutorials demonstrating installation and implementation.

## Design and Program Overview

De-MetaST is written in C++ and is provided as an executable wrapper to include BLAST (De-MetaST-BLAST) as well as an independent executable (De-MetaST). The function of De-MetaST is to implement a search routine based on bitwise comparisons. Initial steps translate the degenerate nucleotide sequences of each primer, as well as their reverse complement sequences, into unique and specific binary representations. This approach facilitates rapid searches of large databases that are also transformed into binary representations. The specific computational steps of De-MetaST are outlined in Figure S1.

**Figure 1:**
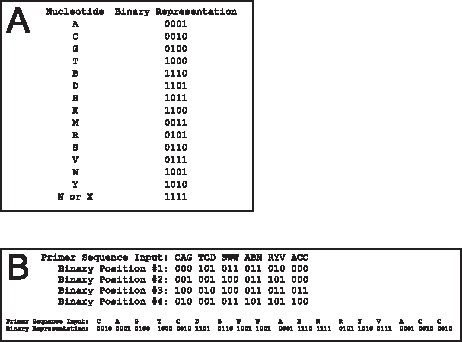
De-MetaST transformation of nucleotide sequences into a binary representation. The binary representation for each of the 16 possible nucleotide character inputs is shown in the upper box. The lower box provides an example of the transformation using a mock primer sequence. Spaced gaps are shown for instructional purposes and do not occur in the De-MetaST search routine.

### How De-MetaST Works

The De-MetaST program initially converts the inputted primer sequences into 4-digit binary code, where the 16 possible combinations of nucleotides include: A, T, C, G, B, D, H, K, M, N (or X), R, S, V, W, and Y (Figure 1). Then, each sequence read within a user defined, FASTA formatted database is converted to 4-digit binary codes and scanned using a bitwise searching operation for the presence of both primer sequences in the appropriate orientation. Limited memory is necessary for this action because each sequence read is individually transformed to binary and immediately scanned for the presence of the primer sequences. The program searches using both the original user inputted primers as well as the reverse and complement of those sequences. This latter search is done to insure identification of target sequences regardless of whether the sense or antisense strand is represented by the database sequence read scanned. The search feature also allows a single primer to serve as both the forward and reverse primer. When primers identify their respective target(s) within a sequence read, the nucleotide sequence delimited by the two primers, termed the *in silico* amplicon, is retrieved. The primer(s) yielding each amplicon are reported in the output. De-MetaST is written to parse *in silico* amplicons >5000 bp into a separate FASTA formatted file that is not subject to BLASTx; users can modify this length restriction by editing the code. All *in silico* amplicons provided in the output represent the sense strand in a 5′ to 3′ orientation. Thus, when positive hits are made to reads representing antisense strands, the complement and reverse of those reads are generated. Any identifying features (*e.g.,* unique read number) as well as the file name for each predicted hit is recovered. Although developed to accept degenerate primers, non-degenerate primers can also be input into De-MetaST. Furthermore, the nucleotide query database(s) themselves may contain sequence reads with degenerate or ambiguous nucleotides (*e.g.,* N). Finally, De-MetaST accepts multiple primer sets as input; the *in silico* amplicons from each set are output into separate FASTA files. As De-MetaST accepts degeneracies in the input primer sequences, it requires absolute conservation in the target sequences; it does not allow for any mismatches between the primer sequence and target. In this way, the user controls the level of primer specificity.

**Figure 2:**
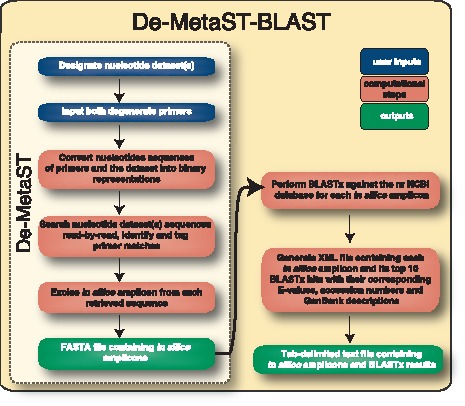
Flowchart outlining De-MetaST-BLAST user actions and corresponding computational processes. Fwd, Forward; Rev, Reverse; NCBI, National Center for Biotechnology Information.

### De-MetaST Paired with BLAST

Once the database sequence files have been queried for predicted PCR amplicons, each *in silico* amplicon is subject to a BLASTx analysis, which translates the nucleotide sequence in all six frames and performs queries for each translation against the non-redundant (nr) NCBI protein database. The top 10 BLASTx hits for each amplicon are formatted as an XML file. The final step of De-MetaST-BLAST compiles all of the meta-information of the BLASTx results for each amplicon retrieved (*e.g.,* hit accession number, E-value, predicted function, nucleotide sequence, database file name, the primer combination that retrieved the amplicon, unique read number) into a single, tab-delimited TXT file. The BLASTx results file can also be exported as an XLS file format for direct use in Microsoft Excel or other suitable program. A graphical overview of the De-MetaST-BLAST workflow is shown in Figure 2.

**Table 1:** *boxB* and 16S rRNA gene *in silico* amplicons identified in representative metagenomes using De-MetaST-BLAST.

CAMERA Metagenome Database Queried	*boxB in silico* amplicons^a^	Unique *boxB* reads^b^	16S rRNA gene *in silico* amplicons^c^	Unique 16S rDNA reads^b^	Database Size [Mbp]	Number of Reads	Average Read Length [bp]^d^	Sequencing Method(s)
CAM_PROJ_FarmSoil.read.fa	2	2	6	6	155	1.38E+05	1117	dideoxysequencing (Sanger)
CAM_PROJ_GOS.read.fa	100	86	3710	965	11598	1.36E+07	915	dideoxysequencing (Sanger) and pyrosequencing (454)
CAM_PROJ_AntarcticaAquatic.read.fa	44	43	4758	1665	23819	6.46E+07	369	dideoxysequencing (Sanger) and pyrosequencing (454)

a The primers *boxB*171F (^5′^ CARGGNGAYACNGARCC ^3′^) and *boxB*265R (^5′^ YTTNCCRTCNCKRTCNGT ^3′^) were used to target an approximately 300 bp region of *boxB*.

b Unique reads were identified using MOTHUR (v.1.27.0) [46].

c The primers 358f (^5′^ CCTACGGGAGGCAGCAG ^3′^) and 517r (^5′^ ATTACCGCGGCTGCTGG ^3′^) [47] were used to target an approximately 190 bp amplicon in the 16S rRNA gene.

d Average read length was estimated by dividing the database size by number of reads. The AntarcticaAquatic database is dominated by pyrosequencing derived reads (98% of all reads), while the GOS dataset is dominated by Sanger derived reads; the exact distribution for GOS reads is not available.

**Figure 3:**
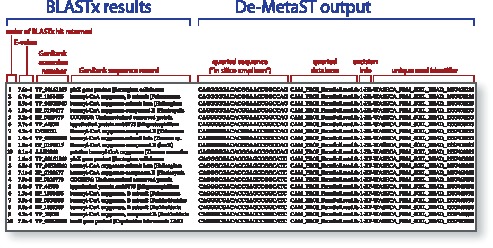
Example of De-MetaST-BLAST output. Text within the box denotes the spreadsheet output for a *boxB* primer set search against the WASECA Farm Soil Metagenome (AAFX01000000) [41] that recovers two *in silico* amplicons. Column descriptors are shown in color; select columns have been truncated due to space constraints. For the “excision info” column, the first alphanumeric character reports the “hit” number within a read (i.e. “1” indicates it is the first *in silico* amplicon found within a single read). The subsequent alphanumeric characters denote the primer orientation yielding the amplicon (F = forward, R = reverse). Whether a unique read identifier is returned is contingent upon the database itself.

## Results and Discussion

We have developed a computational method to generate *in silico* amplifications from degenerate primer sets searched against user defined nucleotide databases. To illustrate the utility of De-MetaST-BLAST, we demonstrate its performance using a novel degenerate primer set designed for use on environmental samples. This primer set targets the bacterial *boxB* gene, which encodes the oxygenase component of a multi-enzyme epoxidase (EC 1.14.13) that is specific to a benzoate catabolic pathway [40]. Three metagenome libraries representing different environments, library size and DNA sequencing methods were searched and found to contain putative *boxB* amplicons of the appropriate size (300 bp) (Table 1). Figure 3 shows the typical output of De-MetaST-BLAST for one of those database searches, which includes for each *in silico* amplicon the top 10 BLASTx hits with their corresponding E-value and GenBank accession number.

**Table 2:** Runtime duration of De-MetaST.

Files Input	Database size [Mbytes]^a^	Sequences indatabase [*10^5^]	Nucleotides inDatabase [Mbp]	Hits	Real Time [s]	User Time [s]	System Time [s]
1	206.1	1.4	154	2	11.7	11.7	0.02
2	412.2	2.8	309	4	23.5	23.4	0.05
3	618.3	4.2	463	6	35.2	35.1	0.07
4	824.4	5.5	618	8	47.6	47.5	0.10
5	1030.5	7.0	772	10	58.6	58.5	0.12
1	206.1	1.4	154	4	11.9	11.9	0.02
2	412.2	2.8	309	8	23.3	23.3	0.05
3	618.3	4.2	463	12	35.6	35.5	0.08
4	824.4	5.5	618	16	47.3	47.1	0.10
5	1030.5	7.0	772	20	58.2	58.0	0.12

a The datasets used for benchmarking were manipulations of the Waseca Farm Soil metagenome (AAFX01000000); the average sequence read length in these datasets is 1117 bp.

To retrieve an *in silico* amplicon, the program requires both primers to match their respective targets in a single sequence read or sequence assembly (contig). Thus, an important consideration in terms of selection of appropriate searchable databases is the average length of the sequence read or assembly contained within it, as well as the desired amplicon size. This concern may be alleviated as longer read sequencing technologies are developed and/or as sequence coverage and assembly algorithms improve. Interestingly, our analysis demonstrates that *in silico* amplicons of ∼300 bp and ∼190 bp, representing *boxB* and 16S rRNA gene amplicons, respectively, can be readily recovered from databases dominated by short read length sequences (*e.g.* AntarcticaAquatic; Table 1). In fact, the 44 *boxB* amplicons derived from the AntarcticaAquatic dataset were found in reads that ranged from 348–541 bp in length. This result suggests that sequence coverage, or depth, is also a contributing factor to *in silico* amplicon recovery. Incidentally, all of the *in silico* amplicons recovered in this demonstration run were found to be homologous to the desired target (E-value ≤1e−4).

In terms of data mining, De-MetaST can provide complementary sequence data for gene diversity studies. As the De-MetaST output provides the sequence from the same genetic positions as that derived from a companion clone library, downstream analysis, such as sequence alignment and subsequent phylogenetic analysis, is streamlined. Thus, *in silico* amplicons retrieved from existing sequence datasets can be readily compared to experimentally derived clone library sequences. Furthermore, as the nucleotide sequences targeted by the primers are returned in the De-MetaST output, users can draw on that information to further refine their primers according to a desired level of functional and/or phylogenetic specificity. The program also has utility beyond searches of environmental sequence databases. It can be used to query any nucleotide dataset, including those derived from single organisms. Thus, it has use in assessing the specificity of primers targeting multi-copy or homologous genes within a single organism or group of organisms.

### Benchmarks and System Requirements

De-MetaST-BLAST has been developed for the long-term support (LTS) Ubuntu operating systems 10.04 LTS and 12.04 LTS. While De-MetaST does not make use of multi-core processors, BLAST maintains that capability. Benchmarks were performed on an Intel i7-2600 processor (3.4 GHz quad-core, 8-thread) desktop using the developed degenerate *boxB* primer set against the Waseca Farm Soil metagenome (AAFX01000000) [41]. This search took approximately 11.7 s (Table 2). When the database size was artificially and incrementally increased up to five-fold (772 Mb) by replication of the original dataset, the processing time remained <1 min. Furthermore, to determine the effect of increased numbers of positive hits on run time, the libraries were seeded with additional sequences containing the target. Doubling of targets within the databases had no effect on run time (Table 2). In contrast to the relatively rapid processing speed of De-MetaST, implementation of the BLAST function can add significant processing time to the process, particularly if a local custom database is used. As an example, for the initial benchmark search against the locally installed Farm Soil metagenome that recovered two hits, the BLASTx function added 39.3 s using two threads. Thus, computational requirements and processing speed are primarily dictated by BLAST. When BLAST is performed remotely–the default setting (see below) –the return time is dependent upon availability and processing speeds of the NCBI servers.

Both De-MetaST and De-MetaST-BLAST can be run on any operating system with a C++ compiler (*e.g.,* standard Windows and Mac OS). However, users would need to ensure the BLAST installation is compatible with their processor.

### Availability of De-MetaST-BLAST

The De-MetaST package and the De-MetaST-BLAST wrapper are made freely available at http://sourceforge.net/p/de-metast-blast/and http://code.google.com/p/de-metast-blast/. These files are also provided as supplemental information to this publication (File S1 and File S2). Along with the program, screencast tutorial videos describe how to install the necessary programs as well as implement the software package with the example dataset provided. The De-MetaST package is self-contained and has no external dependencies, except a C++ compiler, such as g++. De-MetaST-BLAST requires a local BLAST+ suite installation that supports direct query of the NCBI nr protein database using NCBI servers via the –remote option. However, the program can also be configured to query a custom local database. Both approaches are described in tutorial videos provided. Installation of the De-MetaST program is estimated at 5 min, whereas installation of the BLAST+ suite is estimated to take 3 min, excluding download and extraction times, which are dependent on the user’s internet speed and processing power.

### Conclusions

It was recently predicted that the increasing amounts of metagenome sequences will likely serve as a valuable resource in evaluation of the coverage and specificity of previously developed primer sets [42]. De-MetaST-BLAST will provide users with a useful tool in such evaluations. De-MetaST is designed to provide *in silico* amplicons generated by user defined degenerate primers found within a user defined nucleotide database. When paired with BLAST, the program returns the most homologous GenBank hits, which are useful in assessing the specificity of degenerate primers. However, the program does not evaluate PCR kinetics and efficiencies with degenerate primers. Thus, users are encouraged to consult appropriate references on the use and design of degenerate primers (*e*.*g*., [43]–[44]), including those that discuss the merits of utilizing base analogs (*e*.*g*., inosine; [45]) that can reduce the overall degeneracy of primers.

## Supporting Information

Figure S1 **Computational procedures of De-MetaST are illustrated within the De-MetaST-BLAST wrapper.**
(EPS)

File S1 Archive containing the source code for De-MetaST.
(GZ) Click here for additional data file.

File S2 Archive containing the source code for De-MetaST-BLAST.
(GZ) Click here for additional data file.
