# Supplementary material for: De-MetaST-BLAST: A Tool for the Validation of Degenerate Primer Sets and Data Mining of Publicly Available Metagenomes
Source: PLoS One. 2012 Nov 26;7(11):e50362. doi: 10.1371/journal.pone.0050362 (PMC3506598; doi:10.1371/journal.pone.0050362)
Supplement: File S1 — Archive containing the source code for De-MetaST. (GZ) [file pone.0050362.s002.gz › De-MetaST/README-De-MetaST.pdf]

De-MetaST
version 1.0

- To make the executable "De-MetaST", compile with g++ by going into the De-MetaST directory and typing

```
   $ make
```

Input:

1-Forward nucleotide primer (5' to 3')
2-Reverse nucleotide primer (5' to 3')
3-Database file(s)

Output:

1-"De-MetaST_hits.fa" - FASTA formatted file - containing all hit/amplicons ≤5000 bp
2-"non-blasted_De-MetaST_hits.fa" - FASTA formatted file - containing all hit/amplicons >5000 bp

Additional Features:

- Pressing Enter during forward primer prompt will use file named "Primers.txt" as input rather than being prompted for each.
- The Primers.txt file must be located in the same directory as the executable "De-MetaST" file.
- Hit/amplicons >5000 bp by default will not be passed onto BLAST. All retrieved hits >5000 bp are binned into a separate FASTA formatted file "non-blasted_De-MetaST_hits.txt". To change this value, open the "Search.h" file in a simple text editor and locate the statement "int max_length = 5000);" where the 5000 can be changed. The 5000 max length was chosen to avoid exessive computation spent on querying large individual read lines (e.g., each representing a full genome) in BLAST where the feasibility of generating a PCR amplicon is low. Be sure to re-make the De-MetaST executable file if this value is changed.

Re-Making the Executable:

1-Enter into the directory

```
$ cd /home/user/De-MetaST
```

2-Remove the object files and executable

```
$ make clean
```

3-Re-make the executable

```
$ make
```

Example Execution (basic):

- To execute De-MetaST, be sure to navigate to the directory containing the executable before executing below
- In the same directory as the De-MetaST executable, place both "CAM_PROJ_FarmSoil.read.fa" and "Primers.txt"

```
$ ./De-MetaST CAM_PROJ_FarmSoil.read.fa
Input Filename for Primers (press Enter to use Primers.txt)
<PRESS ENTER>
Opening: Primers.txt
Forward Primer: CARGGNGAYACNGARCC
Reverse Primer: YTTNCCRTCNCKRTCNGT

Searching /home/user/CAMERA_Databases/CAM_PROJ_FarmSoil.rea
d.fa
$
```

Example Execution (advanced):

- If your query databases are located in a different directory, you can call to search several of them, each with multiple primer pairs.
- To execute De-MetaST, be sure to navigate to the directory containing the executable before executing below
- Provide the full path to each database queried if they are not located in the same directory as the De-MetaST executable

```
$ ./De-MetaST /home/user/CAMERA_Metagenomes/CAM_PROJ_FarmSo
il.read.fa /home/user/CAMERA_Metagenomes/CAM_PROJ_GOS.read
.fa /home/user/CAMERA_Metagenomes/CAM_PROJ_Antarctica.read
.fa
Input Filename for Primers (press Enter to use Primers.txt)
<PRESS ENTER>
Opening: Primers.txt
Primer Set: 1
Forward Primer: CARGGNGAYACNGARCC
Reverse Primer: YTTNCCRTCNCKRTCNGT

Primer Set: 2
Forward Primer: CCTACGGGAGGCAGCAG
Reverse Primer: ATTACCGCGGCTGCTGG

Searching /home/user/CAMERA_Databases/CAM_PROJ_Antarctica.r
ead.fa
$
```

- Which sequences does your degenerate primer set retrieve?
  - Check in the "De-MetaST_hits.fa"
  - For any >5000 bp amplicons check "non-blasted_De-MetaST_hits.fa"
