# Supplementary material for: De-MetaST-BLAST: A Tool for the Validation of Degenerate Primer Sets and Data Mining of Publicly Available Metagenomes
Source: PLoS One. 2012 Nov 26;7(11):e50362. doi: 10.1371/journal.pone.0050362 (PMC3506598; doi:10.1371/journal.pone.0050362)
Supplement: File S2 — Archive containing the source code for De-MetaST-BLAST. (GZ) [file pone.0050362.s003.gz › De-MetaST-BLAST/README-De-MetaST-BLAST.pdf]

De-MetaST-BLAST
version 1.0

- For the BLASTx feature to work, you *<u>***MUST***</u>* edit the De-MetaST-BLAST.cpp file in a text editor (*e.g.*, gedit or notepad).
- Near the top of the document, locate the comment "//Set BlastDir to the full path to your blastx program." Directly below this statement is where the BLAST directory must point to your specific blastx executable. Be certain to keep the full path (*e.g.*, "/home/user/blast/bin/blastx") surrounded by quotes.

- To make the executable "De-MetaST-BLAST", compile with g++ by going into the De-MetaST-BLAST directory and typing

```
   $ make
```

- Also go into the De-MetaST-BLAST/Table directory to compile the "Table_builder" executable and type

```
   $ make
```

<u>Input</u>:

1-Forward nucleotide primer (5' to 3')
2-Reverse nucleotide primer (5' to 3')
3-Database file(s)

<u>Output</u>:

1-"De-MetaST_hits.fa" - FASTA formatted file - containing all hit/amplicons ≤5000 bp
2-"non-blasted_De-MetaST_hits.fa" - FASTA formatted file - containing all hit/amplicons >5000 bp
3-"BLASTx_results.xml" - XML formatted file - containing all BLAST metadata
4-"De-MetaST-BLAST_functions.txt" - tab delimited file - containing BLASTx predicted functions along with metadata that correspond to the database hit(s) in "De-MetaST_hits.fa"

Additional Features:

- Pressing Enter during forward primer prompt will use file named "Primers.txt" as input rather than being prompted for each. The Primers.txt file must be located in the same directory as the executable "De-MetaST-BLAST" file.
- Hit/amplicons >5000 bp by default will not be passed onto BLAST. All retrieved hits >5000 bp are binned into a separate FASTA formatted file "non-blasted_De-MetaST_hits.fa". To change this value, open the "Search.h" file in a simple text editor and locate the statement "int max_length = 5000);" where the 5000 can be changed. The 5000 max length was chosen to avoid exessive computation spent on querying large individual read lines (e.g., each representing a full genome) in BLAST where the feasibility of generating a PCR amplicon is low.
- The top 10 BLASTx predicted functions are retrieved for each nucleotide hit/amplicon pulled from the query database(s). To change this value, open the "De-MetaST-BLAST.cpp" file in a simple text editor and locate the statement "args[6] = (char *) "10";" where the 10 can be changed.
- By default, in silico amplicons will be sent to NCBI's servers for BLASTx analysis. To run BLASTx locally, open the "De-MetaST-BLAST.cpp" file and locate the statement "bool RemoteBlast = 1;" which should be changed to "bool RemoteBlast = 0;" for local BLASTx to be performed.
- By default, a local BLAST will look for the full nr database. To query a different database (e.g., a manually created one), open the "De-MetaST-BLAST.cpp" file in a simple text editor and locate the statement "#define BlastDatabase "nr"" where the database can be changed to any other database name BLAST will recognize as locally installed. Be certain to keep the name surrounded by quotes.
- The local BLAST uses 8 threads by default. To change this value, open the "De-MetaST-BLAST.cpp" file in a simple text editor and located the statement "args[12] = (char *) "8";" where the 8 can be changed.

Be sure to re-make the De-MetaST-BLAST executable file if changes to any .h or .cpp file are made.

Re-Making the Executable:

1-Enter into the directory

```
$ cd /home/user/De-MetaST-BLAST
```

2-Remove the object files and executable

```
$ make clean
```

3-Re-make the executable

```
$ make
```

<u>Example Execution (basic)</u>:

- If your query database is in the same directory as the De-MetaST-BLAST executable, inputting the full directory path is unnecessary.
- To execute De-MetaST-BLAST, be sure to navigate to the directory containing the executable before executing below

```
$ ./De-MetaST-BLAST CAM_PROJ_FarmSoil.read.fa
Input Filename for Primers (press Enter to use Primers.txt)
<PRESS ENTER>
Opening: Primers.txt
Forward Primer: CARGGNGAYACNGARCC
Reverse Primer: YTTNCCRTCNCKRTCNGT

Searching /home/user/CAMERA_Databases/CAM_PROJ_FarmSoil.rea
d.fa

remote BLASTx running ...
BLASTx finished
$
```

Example Execution (advanced):

- If your query databases are located in a different directory, you can call to search several of them, each with multiple primer pairs.
- To execute De-MetaST-BLAST, be sure to navigate to the directory containing the executable before executing below
- Provide the full path to each database queried if they are not located in the same directory as the De-MetaST-BLAST executable

```
$ ./De-MetaST-BLAST /home/user/CAMERA_Metagenomes/CAM_PROJ_
FarmSoil.read.fa /home/user/CAMERA_Metagenomes/CAM_PROJ_GOS
.read.fa /home/user/CAMERA_Metagenomes/CAM_PROJ_Antarctica.
read.fa
Input Filename for Primers (press Enter to use Primers.txt)
<PRESS ENTER>
Opening: Primers.txt
Primer Set: 1
Forward Primer: CARGGNGAYACNGARCC
Reverse Primer: YTTNCCRTCNCKRTCNGT

Primer Set: 2
Forward Primer: CCTACGGGAGGCAGCAG
Reverse Primer: ATTACCGCGGCTGCTGG

Searching
/home/user/CAMERA_Databases/CAM_PROJ_FarmSoil.read.fa

remote BLASTx running ...
BLASTx finished
$
```

- Does your degenerate primer set retrieve unexpected/undesired targets?
    - Check the predicted functions in the "De-MetaST-BLAST_functions.txt"
    - Right click on "De-MetaST-BLAST_functions.txt" and select a spreadsheet program (e.g., Libre Calc) and indicate it is delimited by tabs.

- Which sequences does your degenerate primer set retrieve?
    - Check in the "De-MetaST_hits.fa"
    - For any >5000 bp amplicons check "non-blasted_De-MetaST_hits.fa"
